# Conversion from mycophenolate mofetil to mizoribine in the early stages of BK polyomavirus infection could improve kidney allograft prognosis: a single-center study from China

**DOI:** 10.1186/s12882-021-02527-3

**Published:** 2021-10-02

**Authors:** Ping Li, Dongrui Cheng, Jiqiu Wen, Xuefeng Ni, Kenan Xie, Xue Li, Jinsong Chen

**Affiliations:** Jinling Hospital, National Clinical Research Center of Kidney Diseases, Medical School of Nanjing University, 305 East Zhong Shan Road, 210002 Nanjing, China

**Keywords:** Mizoribine, BK polyomavirus, BK polyomavirus-associated allograft nephropathy, renal transplantation

## Abstract

**Background:**

Some studies have suggested mizoribine (MZR) could inhibit the replication of BK polyomavirus (BKPyV). The purpose of this study was to explore whether conversion from mycophenolate mofetil (MMF) to MZR in the early stages of BKPyV infection can improve kidney allograft prognosis.

**Methods:**

Twenty-one kidney transplant recipients with BKPyV viruria/viremia and ten with BK polyomavirus-associated allograft nephropathy (BKPyVAN) received MZR conversion therapy were retrospectively identified. The clearance rate of urine and blood BKPyV DNA, change of serum creatinine (SCr), uric acid (UA), hemoglobin (HB), white blood cell (WBC), lymphocyte ratio, platelet (PLT), routine urinalysis, panel reactive antibody (PRA), and gastrointestinal disorders during follow-up of the 2 groups were evaluated and compared.

**Results:**

After MZR conversion therapy, the clearance rate of urine and blood viral load in BKPyV viruria/viremia group were 85.7 and 100 %, while that in BKPyVAN were 40 and 87.5 %, respectively. Stable SCr were observed in all cases of BKPyV viruria/viremia group, while that of BKPyVAN was only 40 % (*P* < 0.001) and one even progressed to end-stage renal disease. The results of routine urinalysis in the two groups showed no significant changes before and after MZR conversion therapy. However, in BKPyV viruria/viremia group, four cases developed acute rejection and one had positive PRA-II but no donor specific antibody, requiring conversion back to MMF. Hyperuricemia was the common adverse effect of MZR.

**Conclusions:**

Conversion from MMF to MZR could help clear BKPyV infection. As compared to BKPyVAN, patients who underwent initiation of MZR conversion therapy in the early stages of BKPyV infection maintained stable allograft function. Prospective studies with larger sample size are needed to ascertain this preliminary finding.

## Background

With the introduction of newer and more potent immunosuppressive agents, the incidence of BK polyomavirus (BKPyV) infection post-kidney transplant increases drastically. BK polyomavirus-associated allograft nephropathy (BKPyVAN) has become a common post-transplant complication.[1–3]Previous studies have shown that up to 30–50 % of kidney transplant recipients developed BKPyV viruria of which approximately 1/3 progressed to viremia and 1–10 % to BKPyVAN. Due to the lack of effective treatment, graft loss in patients with BKPyVAN has been estimated to be as high as 50 %[4].

Mizoribine (MZR), an imidazole nucleoside analog isolated from the mold *Eupenicillium brefeldianum*, is an immunosuppressive agent that has been used extensively in the management of post-transplant immunosuppression and autoimmune diseases[5]. Similar to mycophenolate mofetil (MMF), MZR inhibits cellular and humoral immune responses by blocking inosine 5-monophosphate dehydrogenase, which is a rate-limiting enzyme for de novo purine synthesis and critical for the proliferation of T and B lymphocytes[6]. Moreover, MZR also can inhibit lymphocyte proliferation via affection guanosine monophosphate synthetase[7]. Although the pharmacological efficacy of MZR against lymphocyte proliferation was weaker than MMF, conversion from MMF to MZR associated with significantly fewer episodes of leukopenia, gastrointestinal disorder, and especially cytomegalovirus (CMV) infection while preventing rejection to some extent in renal transplantation[8–10]. During the conversion from MMF to MZR, the lower risk of virus infection might be associated with the reduced intensity of immunosuppression, which facilitates the immune system to kill the virus. Significantly, in vitro and in vivo studies showed that MZR could inhibit the replication of CMV, hepatitis C virus, and foot-and-mouth disease virus [11–13]. Previous studies have also suggested conversion from MMF to MZR correlated with lower BKPyV viruria/viremia[14, 15]. Nevertheless, the efficacy and safety profiles of MZR for BKPyVAN remains uninvestigated, to the best of our knowledge. Therefore, the objective of the current study was to explore whether conversion from MMF to MZR in the early stages of BKPyV infection can improve kidney allograft prognosis.

## Methods

### Study subjects

From November 2015 to June 2018, kidney transplant recipients with BKPyV viruria/viremia or biopsy-proven BKPyVAN who sought medical attention at the Jinling Hospital, Medical School of Nanjing University were retrospectively identified by searching the electronic medical records. We then further scrutinized those who received MZR treatment and whose follow-up data were complete among the identified patients. Patients without regular testing for urine/blood BKPyV DNA, concomitant acute allograft rejection, or those with an eGFR < 30ml/min/1.73m^2^ were excluded from final analysis. Informed consent was obtained from all patients, and the study protocol was approved by the Human Subjects Committee of Jinling Hospital (Nanjing, China).

## Measurements and definitions

The collected information included the patient’s gender, age, etiologies of end stage renal decease(ESRD), the type of allograft, post-transplant time, urine/blood BKPyV burden, preoperative induction therapy, baseline immunosuppressive regimens, clinically/pathologically documented delayed graft function (DGF) or acute rejection, serum creatinine (SCr), uric acid (UA), hemoglobin (HB), white blood cell (WBC), lymphocyte ratio, platelet (PLT), tacrolimus (Tac) trough level, routine urinalysis, the number of human leukocyte antigen(HLA) mismatch, and the panel reactive antibody (PRA) levels at baseline and during follow-up after MZR conversion treatment. In addition, the presence of gastrointestinal disorders after conversion therapy was obtained from the electronic case system or patients self-report.

Acute rejection was diagnosed based on kidney allograft biopsy findings or clinical diagnosis. DGF was defined as anuria, oliguria or SCr > 400µmol/L or continuous renal replacement therapy was needed occurred within a week post-kidney transplant. The Chronic Kidney Disease Epidemiology equation was applied to calculate the eGFR. ESRD was defined as eGFR < 15ml/min/1.73 m². Stable kidney allograft function was defined as SCr increased ≤ 20 % from baseline.

## Screening and quantification of BKPyV DNA

The regular testing protocol for urine/blood BKPyV DNA posttransplant at our center was mainly based on the American Society of Transplantation infection guideline[1]. Monthly urine/blood BKPyV DNA testing for the first 3 months, every 3 months until 2 years posttransplant, and then once a year. If detectable, followed by biweekly testing for follow-up and decision making. BKPyV DNA quantification was carried out using the BKPyV nucleic acid quantitative detection kit (SinoMD, China) with an ABI Prism 7500 Fast Renal Time PCR System (Applied Biosystems, America). The minimal detection threshold of BKPyV DNA was 1 × 10^3^ copies/mL, which was employed to denote BKPyV viruria or viremia. High-level viruria was defined as urine BKPyV DNA ≥ 10^7^ copies/ml.

## Pathologic diagnosis of BKPyVAN

**Fig. 1:**
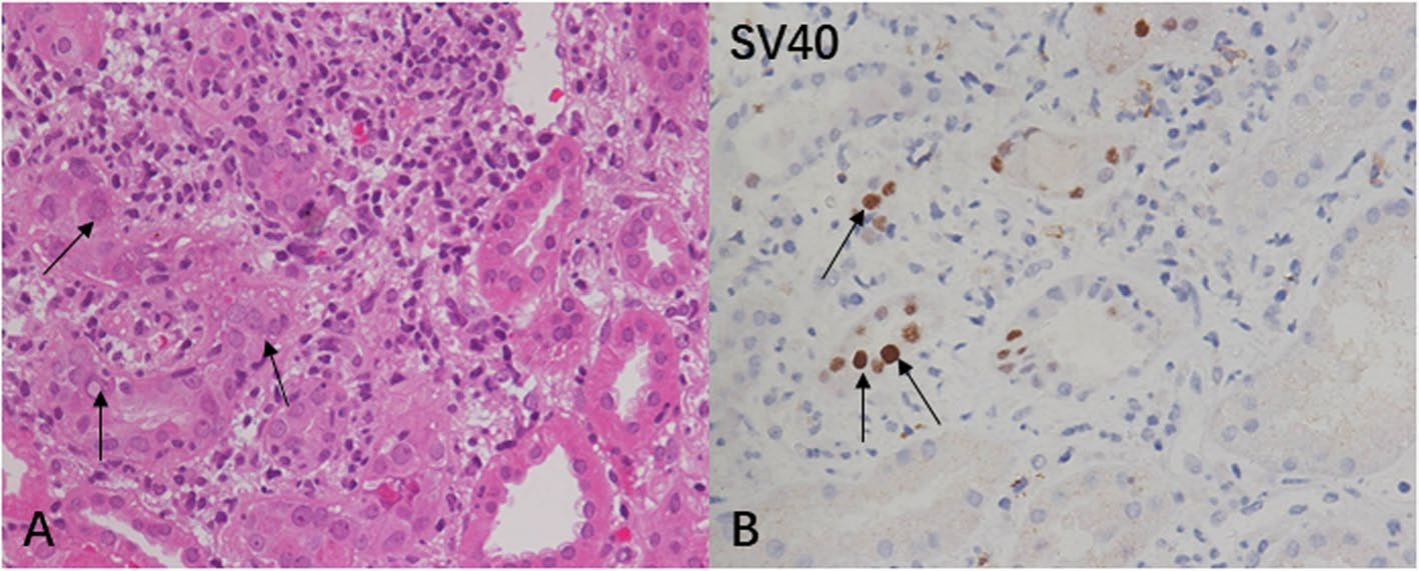
**Histopathological features of BK polyomavirus-associated allograft nephropathy (**BKPyVAN**)**. **(A**) Light microscope image of BKPyVAN. The histological manifestations are characterized by nuclear inclusion bodies in tubular epithelial cells (arrow, Hematoxylin-Eosinstained paraffin section, ×400). (**B**) Immunostaining of BK polyomavirus-infected cells with anti-SV40 large T antigen antibodies showing the nuclei of renal tubular epithelial cells have a transparent center and thorn-shaped periphery (arrow indicates the immunohistochemical staining, ×400)

In line with the most recent version of the American Society of Transplantation infection guidelines[1], BKPyVAN was diagnosed and staged base on detecting histopathologic signs of viral cytopathic changes (intranuclear viral inclusions in tubular epithelial cells and/or Bowman’s capsular epithelial cells), accompanied by tubular epithelial cells necrosis and denudation of basement membranes, as well as tubule-interstitial infiltrates and tubulitis (Fig. 1 A). The diagnosis of BKPyVAN was further confirmed by positive SV40 staining (Fig. 1B). The Banff score of tubular and interstitial lesions with reference to Banff 2017[16].

## Induction therapy

Antithymocyte globulin or basiliximab was used for induction therapy in renal transplantation. Antithymocyte globulin was intravenously injected at a dose of 1 mg/kg/day during the transplantation and on the first two days post-transplantation, while basiliximab was intravenously injected at a dose of 20 mg/day on Day 0 and Day 4 post-transplantation. Additionally, methylprednisolone (500 mg/day, intravenously) was given to all patients from Day 0 to Day 2 post-transplantation. The method and dose of induction therapy were determined by the immunological risk (such as HLA mismatch and PRA) and infection risk of patients.

## Baseline immunosuppressive regimens

The post-transplant maintenance immunosuppressive regimens in all patients were consisted of Tac, MMF and prednisone (Pred). Tac was started at 0.15 g/kg/d in 2 divided doses, targeting whole blood through levels of 6–10 ng/ml within 6 months. Progressive reduction of Tac was started from 6 + month, to reach target levels of 5–8 ng/ml through months + 6 to 12, and 4-6ng/ml thereafter. MMF was started at a dosage of 0.75 g twice daily. Pred was started at 80 mg/d from postoperative day 3, reduced 10 mg daily to maintenance dosages of 20 mg/d, then gradually reduced to 10-15 mg/d at post-transplant month 6, 5 mg/d at post-transplant month 12 and maintained thereafter.

## Protocol of conversion to MZR

For patients with high-level BKPyV viruria and/or BKPyV viremia, MMF was switched to MZR (200 mg/d). Urinary and serum BKPyV DNA were measured every two weeks, and if the urine/blood BKPyV DNA decreased, followed by testing every 1 to 3 months for follow-up and decision making after MZR conversion.

### Statistical analysis

Statistical analyses were conducted using SPSS (v25.0, SPSS, Chicago, IL) software. Continuous variables with normal distribution were presented as the mean ± standard deviation and compared using the Student *t*-test, whereas those with non-normal distribution were expressed as the medians (quartiles) and compared with the Mann-Whitney U test. Categorical variables were expressed as percentages and compared using Pearson chi-square (or Fisher’s exact test) with the Bonferroni correction for *P* values. A two-sided *P* value < 0.05 was considered statistically significant.

## Results

### Baseline patient characteristics

As shown in Table 1, data from 21 patients with BKPyV viruria/viremia and 10 with BKPyVAN were finally analyzed. The 2 groups showed no significant differences with regard to patient demographics, the type of allograft, number of HLA mismatch, documented DGF or acute rejection, use of immune induction therapy, baseline immunosuppressive regimens, Tac trough level, SCr, UA, WBC, lymphocyte ratio, HB, PLT and PRA. Although BKPyV viruria was observed in all cases in both groups, urine BKPyV load in the BKPyV viruria/viremia group was significantly lower than that in the BKPyVAN group (8.62 vs. 10.16 log10copies/ml, *P* = 0.005). The proportion of viremia was significantly lower in BKPyV viruria/viremia group than that in the BKPyVAN group (14.3 % vs. 80 %, *P* = 0.001).

**Table 1:** Baseline characteristics of patients

Demographic	BKPyV viruria and (or) viremia (*n* = 21)		BKPyVAN (*n* = 10)	*P* value
Male, n (%)	15(71.4)	7(70.0)		0.675
Age(years)	34.6 ± 11.1	37.0 ± 10.3		0.564
Etiologies of end stage renal disease				-
IgA nephropathy, n (%)	4(19.0)	0		
Membranous nephropathy, n (%)	0	1(10.0)		
Focal Segmental Glomerulosclerosis, n (%)	0	2(20.0)		
Lupus nephritis, n (%)	1(4.8)	0		
Unknow, n (%)	16(76.2)	7(70.0)		
Type of allograft				0.880
Living-related donor kidney, n (%)	9(42.9)	4(40.0)		
Deceased-related donor kidney, n (%)	12(57.1)	6(60.0)		
Number of HLA mismatch, n	4.7±1.7	5.2±1.7		0.435
Post-transplantation time(months)	6.7 ± 8.7	11.5 ± 10.3		0.187
Viruria, n (%)	21(100.0)	10(100.0)		1.000
Mean (Log10, copies/ml)	8.62 ± 1.28	10.16 ± 1.35		0.005
Viremia, n (%)	3(14.3)	8(80.0)		0.001
Mean (Log10, copies/ml)	4.65 ± 0.95	4.51 ± 0.85		0.821
PRA positive, n (%)	1(4.8)	0		1.000
SCr (mg/dl)	1.51 ± 0.42	1.79 ± 0.37		0.083
UA (umol/L)	366.3 ± 79.2	434.5 ± 112.7		0.068
WBC(x10^9/L)	7.98 ± 1.63	7.72 ± 3.9		0.801
Lymphocyte ratio (%)	27.52 ± 8.2	25.68 ± 7.63		0.559
HB (g/L)	126.9 ± 21.3	109.8 ± 38.6		0.127
PLT(x10^9/L)	237.6 ± 79.8	194.8 ± 66.0		0.155
Flow-up time (months)	15.3 ± 11.2	22.4 ± 5.9		0.074
DGF, n (%)	0	1(10.0)		1.000
Acute rejection, n (%)	1(4.8)	0		1.000
Intraoperative induction therapy, n (%)				0.575
Antithymocyte globulin	6 (28.6)	3 (30.0)		
Basiliximab	10 (47.6)	3 (30.0)		
Unknowing	5 (23.8)	4 (40.0)		
Baseline immunosuppression regimens				
Tac + MMF + Pred	21(100.0)	10 (100.0)		1.000
Tac trough level(ng/ml).	7.1±1.9	6.6±1.8		0.439

*BKPyV* BK polyomavirus; *BKPyVAN* BK polyomavirus associated allograft nephropathy; *DGF* delayed graft function; *HB* hemoglobin; *HLA* human leukocyte antigen; *MMF* mycophenolate mofetil; *PLT* platelet; *Pred* prednisone; *PRA* panel reactive antibody; *Scr* serum creatinine; *Tac* tacrolimus; *UA* uric acid; *WBC*: white blood cell

Additionally, the histological stages of BKPyVAN and Banff score of tubular and interstitial lesions of all patients in BKPyVAN group were shown in Table 2.

**Table 2:** Histological stages of BKPyVAN and Banff score of tubular and interstitial lesions

Cases	Histological stages	Banff score			
		Inflammation (i)	Tubulitis (t)	Tubular atrophy (ct)	Interstitial fibrosis (ci)
1	B1	i1	t2	ct1	ci1
2	A	i1	t1	ct1	ci1
3	B3	i3	t2	ct2	ci2
4	B1	i1	t3	ct1	ci1
5	B1	i1	t2	ct2	ci2
6	B3	i3	t3	ct1	ci1
7	C	i1	t1	ct3	ci3
8	B2	i2	t2	ct2	ci2
9	A	i1	t1	ct1	ci1
10	B2	i2	t1	ct2	ci1

*BKPyVAN* BK polyomavirus associated allograft nephropathy

## Changes of BKPyV DNA loads

**Fig. 2:**
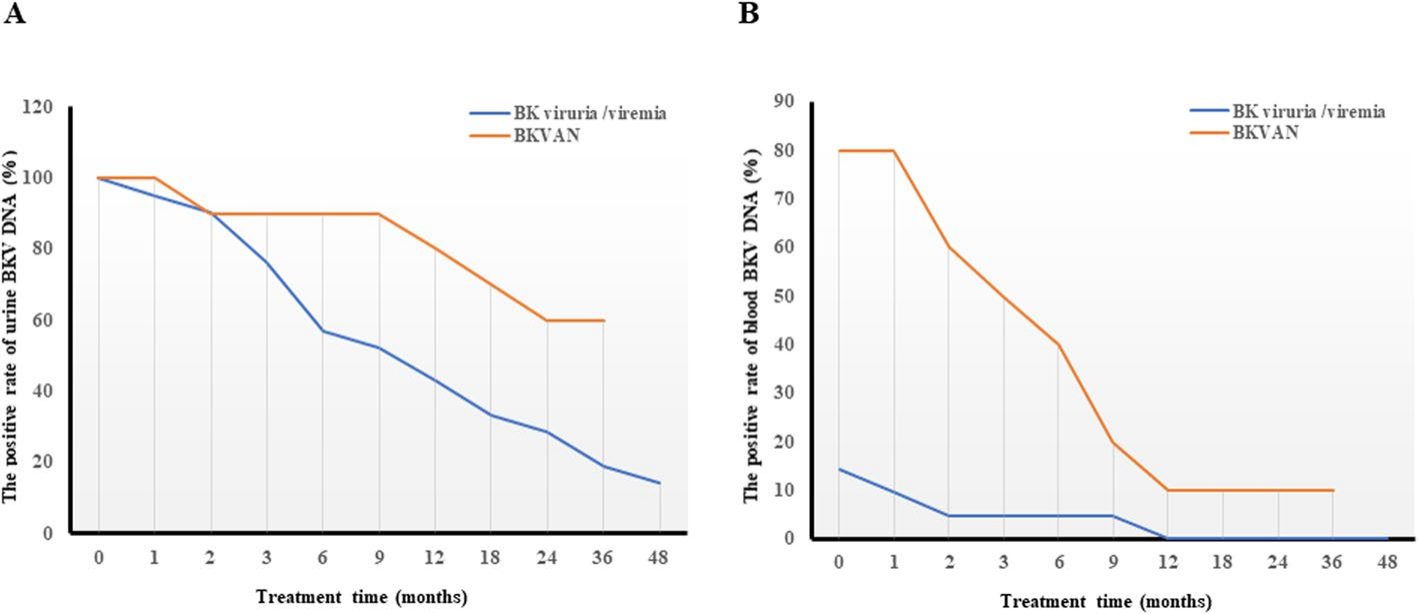
**Changes of the urine and blood BK polyomavirus (BKPyV) DNA positive rate after mizoribine conversion therapy.** (**A**) Changes of the urine BKPyV DNA positive rate. The urine BKPyV DNA positive rate were decreased in 2 groups, especially the BKPyV viruria/viremia group. The negative conversion rate of urine viral load in BKPyV viruria/viremia group and BK polyomavirus-associated allograft nephropathy (BKPyVAN) group were 85.7 and 40 %, respectively. (**B**) Changes of the blood BKPyV DNA positive rate. The blood BKPyV DNA, DNA positive rate was significantly decreased in the 2 groups and the negative conversion rate of blood viral load in BKPyV viruria/viremia group and BKPyVAN group were 100 and 87.5 %, respectively

The mean follow-up time after MZR conversion for patients with BKPyV viruria/viremia group and BKPyVAN group were 15.3 and 22.4 months respectively. BKPyV DNA load in both the urine and blood were decreased in all cases in both groups. The negative conversion rate of urine viral load in BKPyV viruria/viremia group was significantly higher than that in BKPyVAN group (85.7 % vs. 40 %, *P* = 0.015) (Fig. 2 A), but there was no significant statistical difference in those of blood viral load between the 2 groups (100 % vs. 87.5 %, *P* = 1.000) (Fig. 2B).

## Renal allograft function

**Fig. 3:**
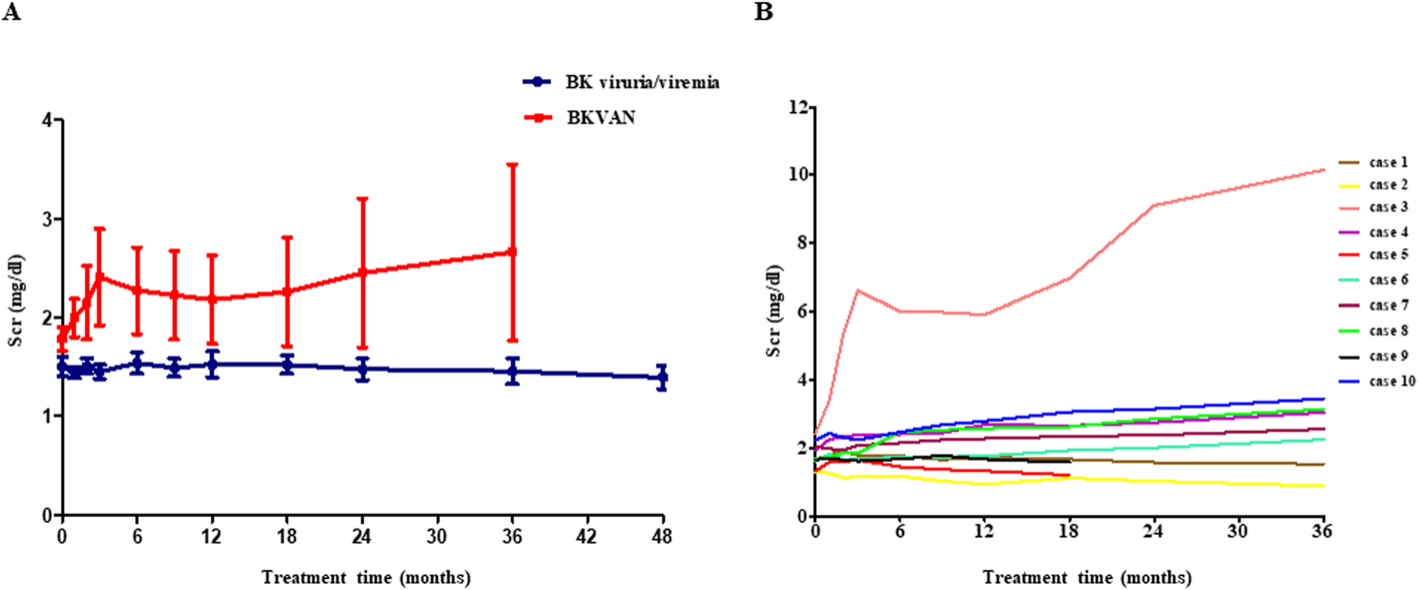
**Changes of the serum creatinine (SCr) after mizoribine conversion therapy.** (**A**) a stable SCr was observed in BK polyomavirus (BKPyV) viruria/viremia group, while that of BK polyomavirus-associated allograft nephropathy (BKPyVAN) group was increased progressively. **(B)** There were only 4/10(40 %) patients (case2, 5, 7, 9) in BKPyVAN group had a stable SCr, while the rest of the patients had a progressive increase SCr and one (case 3) even progressed to end-stage renal disease

During the follow-up period, a stable SCr was observed in all patients (100 %) in BKPyV viruria/viremia group, while that of BKPyVAN was only 4/10(40 %) (*P* < 0.001). All the rest of the patients in BKPyVAN had a progressive increase SCr and one even progressed to end-stage renal disease (Fig. 3 A-B). In addition, although we didn’t monitor the 24-hour urinary protein quantitative of patients, the results of routine urinalysis in the two groups showed no significant changes before and after MZR conversion therapy (data were not shown).

## MZR Safety

**Fig. 4:**
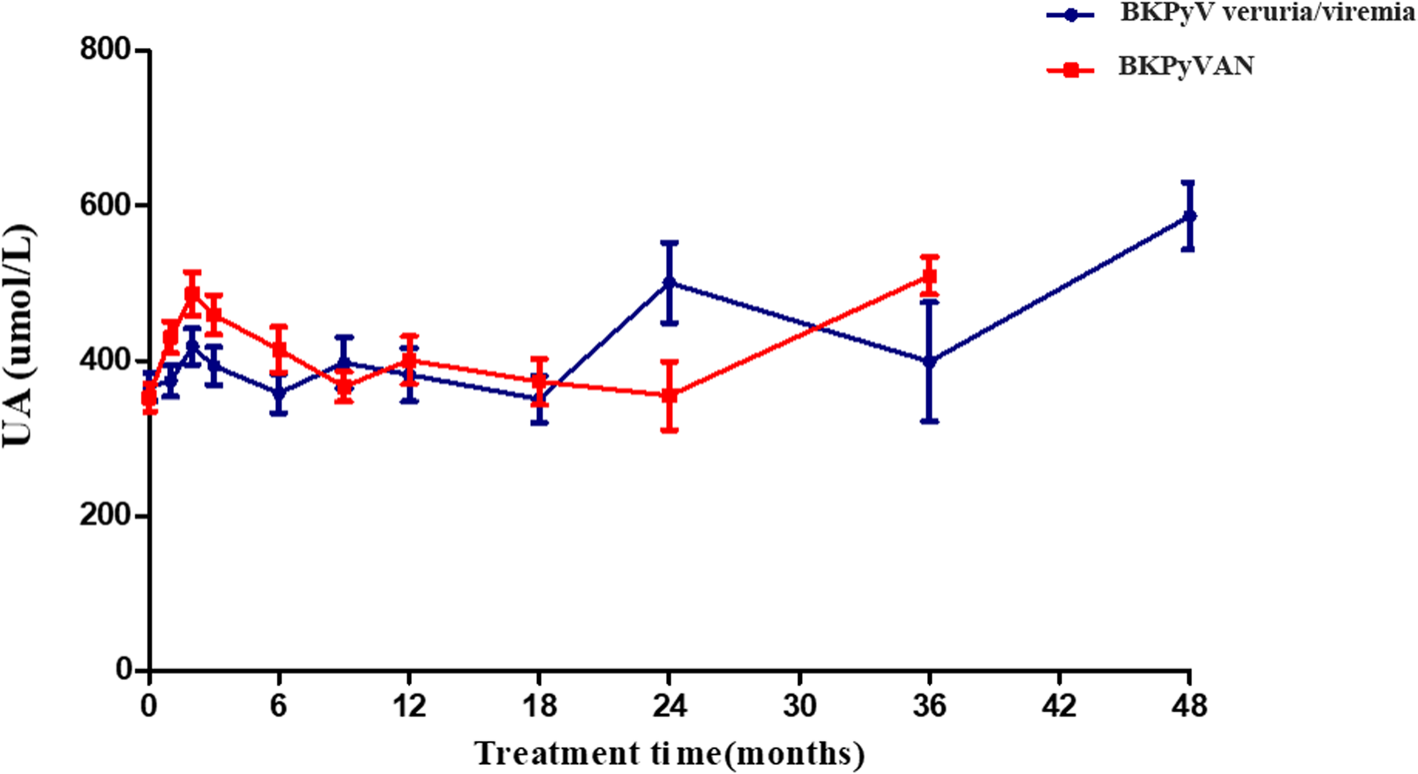
**Changes of the blood uric acid (UA) after mizoribine (MZR) conversion therapy.** The UA of the BK polyomavirus (BKPyV) viruria/viremia group and BK polyomavirus-associated allograft nephropathy (BKPyVAN) group were increased after MZR treatment and was easily controlled by uric-acid-lowering drugs such as benzbromarone, or febuxostat

**Fig. 5:**
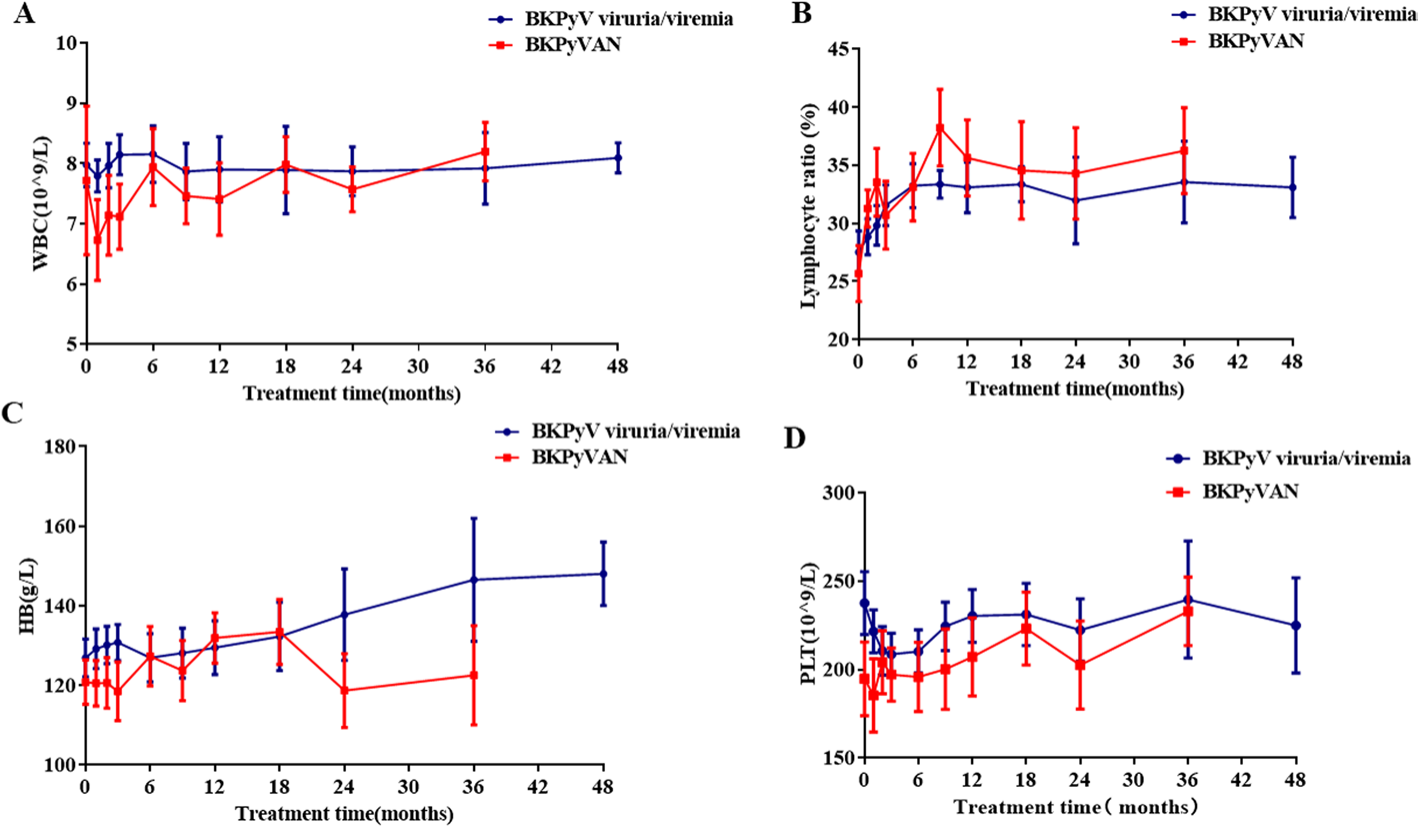
**Changes of the hematologic parameters after mizoribine (MZR) conversion therapy.** The white blood cell (WBC) count (**A**), lymphocyte ratio (**B**), hemoglobin (HB) (**C**) and blood platelet (PLT) (**D**) of the BK polyomavirus (BKPyV) viruria/viremia group and BK polyomavirus-associated allograft nephropathy (BKPyVAN) group were stable after MZR treatment

Increased UA levels seen in the 2 groups showed no statistical significance and was easily controlled by uric-acid-lowering drugs such as benzbromarone, or febuxostat. (Fig. 4). No gastrointestinal disorders were observed in both groups. Hematologic parameters, such as WBC, lymphocyte ratio, HB and PLT showed no significant changes before and after MZR treatment (Fig. 5 A-D). In BKPyV viruria/viremia group, four cases developed acute rejection at 6 months, 6 months, 9 months, and 18 months after MZR conversion, respectively. but all the PRA of which were negative. Three cases were performed kidney transplant biopsy and the Banff diagnosis were T cell-mediated rejection, one of which presented with SCr increase along with elevation of blood pressure and weight gain was performed methylprednisolone pulse therapy (500 mg/d, 3 days) and switched MZR to MMF (0.75 g, twice daily). The other three cases presented with elevation of SCr, increased from 1.34 mg/dl to 1.42 mg/dl, 1.45 mg/dl to 1.67 mg/dl, and 1.31 mg/dl to 1.52 mg /dl, respectively, were all switched MZR to MMF (0.75 g in the morning, 0.5 g in the evening). All of them were reversed after timely treatment. In addition, one had positive PRA-II (DP2 and DP5, the median fluorescence intensity was 1390 and 1413, respectively) but no donor specific antibody (DSA, HLA genotyping of donor were A2/A11, B13/B13, DQ5/DQ7, DR12/DR15). In the BKPyVAN group, none of the patients developed rejection or PRA positive after MZR conversion therapy.

## Discussion

BKPyVAN has been one of the major causes of renal allograft dysfunction and even graft loss[1]and the main treatment option is to reduce or discontinue immunosuppressive agents, with a risk of secondary acute rejection[17]. Most patients had no obvious clinical symptoms during the stage of BKPyV viruria/viremia, leading to frequent missed diagnosis and delayed treatment. When patients underwent indication biopsies, most of them had already progressed to BKPyVAN with poor response to treatment that was accompanied deterioration of renal allograft function. Therefore, early monitoring, diagnosis and treatment of BKPyV infection bears importance to effectively delay BKPyVAN progression and graft function deterioration.

The anti-BKPyV effect of MZR was first reported by Funahashi et al., whose group observed that urine BKPyV DNA decreased or even turned to negative within 12 months after conversion to MZR from a baseline BKPyV DNA level of 2.2x10^2^ to 5.5x10^6^ copies/mm^3^. More importantly, no acute rejection or graft function deterioration occurred during the administration of MZR[14]. In a prospective study involving 50 kidney transplant recipients with high-level BKPyV viruria (including 11 with concomitant BK viremia) after 6 months of MZR therapy, Yuan et al. found that the clearance rate of BKPyV viremia was 100 % and only 3 (6 %) patients still had high-level BKPyV viruria [15]. Nevertheless, all these studies were focused on patients with BKPyV viruria or viremia. There were no relevant studies on BKPyVAN, and whether MZR treatment at different stages of BKPyV infection had different impact on kidney allograft long-term prognosis remained unclear.

In the present study, the mean follow-up time of patients with the BKPyV viruria/viremia group and BKPyVAN group were 15.3 and 22.4 months, respectively. The urinary and serum BKPyV DNA were significantly decreased in all cases, especially the BKPyV viruria/viremia group. Our study corroborated that MZR could inhibit BKPyV and even BKPyVAN. Previous studies have demonstrated that the antiviral activity of MZR involves inhibition of Inosine-5′-monophosphate dehydrogenase (IMPDH), an essential enzyme for the synthesis of guanosine monophosphate from inosine monophosphate through de novo pathway and its inhibition can lead to depletion of intracellular GTP pools[12, 13, 18]. Therefore, the mechanism of MZR against BKPyV may be also involve inhibition of IMPDH, further in Vitro and in Vivo experiments are needed. Additionally, during the follow-up period, the SCr in all patients in the BKPyV viruria/viremia group remained stable, but increased progressively in most of the cases in BKPyVAN group and one even progress to ESRD, considering related to the later pathological stage (Table 2). Numerous viru in BKPyVAN group have directly damaged the renal tubulointerstitial tissue and the inflammatory response secondary to BKPyV infection further aggravated the graft injury, eventually leading to irreversible graft dysfunction. Our previous study found that with increasing stages of BKPyVAN, the numbers of inflammatory cells infiltration were significantly increased[19]. Therefore, the results suggested MZR conversion therapy should be given in the early stage of BKPyV infection, namely BKPyV viruria and /or viremia, in order to effectively delay the progression of renal allograft function.

In congruent with earlier studies, hyperuricemia was noted to be the most common adverse effect of MZR[9]. This may suggest that MZR interfere with purine metabolism. A multicenter study reported that secondary hyperuricemia correlated with MZR blood concentration[5]. Furthermore, as MZR is excreted by the kidneys, its blood concentrations are largely dependent on renal allograft function. Hence, MZR blood levels and blood uric acid should be monitored biweekly for the first one months and then every 3 months, and the dosage adjusted accordingly during treatment course. A retrospective analysis reported that the incidence of gastrointestinal symptoms and leukopenia were significantly lower in those treated with MZR than with MMF[20]. In line with this, no gastrointestinal or hematologic side effects were observed in this study. Additionally, there were 4 cases of acute rejection and 1 of positive PRA in BKPyV viruri/viremia group, while there no patients developed acute rejection or positive PRA in BKPyVAN group during MZR conversion, considering the difference might be related to the lower immunity in patients with BKPyVAN given the same drug-switching therapy. Although all of patients with acute rejection reversed after timely treatment, regular monitoring of renal function and PRA were necessary after switching to MZR from MMF for decision making timely. Because acute rejection was also diagnosed based on clinical diagnosis besides kidney allograft biopsy in our study, the occurrence of rejection in our study higher compared to previous study. However, there were smaller sample included in previous study and the results of the study need to be further verified by expanding the sample [14].

However, the present study is subjected to several limitations. This study is retrospective and we didn’t routinely monitor MZR blood concentration in all patients. Thus, the optimal MZR blood concentrations remain to be investigated. Secondly, repeated renal graft biopsy didn’t performe on BKPyVAN patients after MZR conversion therapy, so we couldn’t observe the pathological changes of renal graft tissue. Thirdly, the withdrawal of MMF may have certain impact on the clearance of BKPyV and large sample randomized controlled trials are needed. Finally, the sample was small and prospective studies with larger sample size are needed to ascertain this preliminary finding.

## Conclusions

Our study demonstrated that conversion from MMF to MZR could help clear BKPyV infection. Compared to patients with BKPyVAN, patients who underwent initiation of MZR conversion therapy in the early stages of BKPyV infection maintained stable allograft function. Hyperuricemia remains the most common adverse effect of MZR. Prospective studies with larger sample size are needed to ascertain this preliminary finding.

***Author information***.

^1^National Clinical Research Center of Kidney Diseases, Jinling Hospital, Medical School of Nanjing University, 305 East Zhong Shan Road, Nanjing 210,002, China.

## Data Availability

The datasets used and/or analyzed during the current study are available from the corresponding author on reasonable request.

## Abbreviations

BKPyV: BK polyomavirus
BKPyVAN: BK polyomavirus-associated allograft nephropathy
DGF: Delayed graft function
ESRD: End stage renal disease
HB: Hemoglobin
HLAHuman leukocyte antigen: Human leukocyte antigen
MMF: Mycophenolate mofetil
PLT: Platelet
Pred: Prednisone
PRA: Panel reactive antibody
SCr: Serum creatinine
Tac: Tacrolimus
UA: Uric acid
WBC: White blood cell
